# New generation ICG-based contrast agents for ultrasound-switchable fluorescence imaging

**DOI:** 10.1038/srep35942

**Published:** 2016-10-24

**Authors:** Shuai Yu, Bingbing Cheng, Tingfeng Yao, Cancan Xu, Kytai T. Nguyen, Yi Hong, Baohong Yuan

**Affiliations:** 1 Ultrasound and Optical Imaging Laboratory, Department of Bioengineering, The University of Texas at Arlington, Arlington, TX 76019, USA; 2 Joint Biomedical Engineering Program, The University of Texas at Arlington and The University of Texas Southwestern Medical Center at Dallas, TX 75390, USA; 3 Department of Bioengineering, The University of Texas at Arlington, Arlington, TX 76019, USA

## Abstract

Recently, we developed a new technology, ultrasound-switchable fluorescence (USF), for high-resolution imaging in centimeter-deep tissues via fluorescence contrast. The success of USF imaging highly relies on excellent contrast agents. ICG-encapsulated poly(N-isopropylacrylamide) nanoparticles (ICG-NPs) are one of the families of the most successful near-infrared (NIR) USF contrast agents. However, the first-generation ICG-NPs have a short shelf life (<1 month). This work significantly increases the shelf life of the new-generation ICG-NPs (>6 months). In addition, we have conjugated hydroxyl or carboxyl function groups on the ICG-NPs for future molecular targeting. Finally, we have demonstrated the effect of temperature-switching threshold (T_th_) and the background temperature (T_BG_) on the quality of USF images. We estimated that the T_th_ of the ICG-NPs should be controlled at ~38–40 °C (slightly above the body temperature of 37 °C) for future *in vivo* USF imaging. Addressing these challenges further reduces the application barriers of USF imaging.

Near-infrared (NIR) fluorescence imaging in opaque biological tissue based on diffused photons provides the tissue’s structural, functional, and molecular information with high sensitivity via non-ionizing radiation^1^,^2^,^3^. NIR light can also penetrate tissue by several centimeters and enable multiplex imaging via different wavelengths^1^,^2^,^3^. Unfortunately, as a result of the tissue’s high light scattering and attenuation, its poor spatial resolution prevents it from resolving microscopic information in deep tissues. Although fluorescence diffuse optical tomography (FDOT) takes advantage of highly scattered photons with reconstruction algorithms and thus could achieve an image depth of a few centimeters, the resolution is still low (a few millimeters)^4^,^5^,^6^,^7^. However, it is highly desirable to achieve fluorescence imaging with high resolution in deep tissue because many physiological and pathological micro-phenomena may locate in tissue at a depth (or thickness) of centimeters (from a surface where an instrument can reach), such as microcirculation, angiogenesis, and cancer metastasis^2^,^8^,^9^.

Recently, to achieve this goal, several technologies have been developed by using focused ultrasound to confine fluorescence emission into a small volume. Thus, fluorescence images with acoustic resolution (tens or hundreds of microns depending on the frequency) or even better can be achieved in deep tissues^10^,^11^,^12^,^13^,^14^,^15^,^16^,^17^,^18^,^19^,^20^,^21^,^22^,^23^. Time-reversed ultrasonically encoded optical focusing aims to focus the excitation light into deep tissue and to achieve acoustic resolution^10^,^11^,^12^,^13^,^14^. On the other hand, the ultrasound modulated or switchable fluorescence method aims to confine fluorescence emission into the ultrasound focal volume to achieve high resolution^15^,^16^,^17^,^18^,^19^,^20^. The advantage of the former is that existing fluorophores can be directly used without any modification. The disadvantage is that the system is very complicated because optical phase conjugation technique is adopted. In addition, the dynamic response speed and the sensitivity or signal-to-noise ratio (SNR) in deep tissue should be further improved. In contrast, the latter usually adopts simple system by using a focused ultrasound wave to control fluorescence emission. However, to achieve high sensitivity or SNR, the contrast agents usually need to be specifically designed to significantly minimize background fluorescence emission from the fluorophores.

Among these technologies, we recently developed ultrasound-switchable fluorescence (USF), which belongs to the latter^15^,^16^,^24^,^25^. The performance of USF depends on the performance of its contrast agent. In USF, background fluorescence from the agents out of the ultrasound focal volume is maximally suppressed to minimize the noise. The fluorescence emission from the switched-on agents in the ultrasound focal volume is strong to achieve high signal^15^. In USF imaging, there are two key components: an excellent USF contrast agent and a sensitive USF imaging system. When a high intensity focused ultrasound (HIFU) pulse is applied, contrast agents in the focal volume can be thermally switched on and emit fluorescence while contrast agents outside the focus remain off. Scanning the ultrasound focus can produce a high-resolution USF image. Because both the delivery of the excitation light and the collection of emission light depend on highly scattered (or diffused) NIR photons, USF can achieve an imaging depth of a few centimeters.

The quality of a USF image depends significantly on the quality of the contrast agent^15^,^16^,^24^,^25^. Therefore, this study aims to upgrade (so-called new generation) one of our previously developed NIR USF contrast agents: indocyanine green (ICG)-encapsulated poly(N-isopropylacrylamide) (PNIPAM) nanoparticles (ICG-NPs)^15^,^24^. ICG-NP is a type of thermo-sensitive USF contrast agent that responds to the ultrasound-induced temperature change in the focal volume. Our previous publications have successfully demonstrated a family of ICG-NPs as an excellent USF contrast agent^15^,^25^. The ICG-NP outstands in the followings. First, ICG is an FDA-approved and commercially available NIR fluorophore. Thus, it is relatively safe and cheap, and synthesizing USF contrast agents is relatively easy (no extra work is needed to synthesize fluorophores), which may increase the wide use of USF imaging. In addition, auto-fluorescence can be efficiently minimized because the excitation wavelength can be selected from 780 to 810 nm. Second, ICG-NP provides an acceptable on-to-off ratio of fluorescence intensity (I_ON_/I_OFF_ = ~3–9) and therefore a reasonable signal-to-noise ratio (SNR)^15^,^24^. Third, the temperature threshold at which to switch on ICG (T_th_, i.e., the low critical solution temperature (LCST)) can be accurately controlled from room temperature (for *in vitro* studies) to the one above body temperature (>37 °C for *in vivo* studies). Fourth, the temperature transition bandwidth (T_BW_) is narrow (generally ~5 °C); thus the switching efficiency is high^15^,^24^.

However, our previously developed ICG-NP (so-called first-generation ICG-NP) is also limited^15^. First, the shelf life (in terms of the temperature-switching property) is less than 1 month (stored at 4 °C) and thus it is difficult to use for a long-term study. Second, first-generation ICG-NP is lack of functional groups (such as carboxyl or amino) on its nanoparticle. Thus, the first-generation ICG-NP is limited for molecular imaging. In this study, we developed two new protocols that differ from the previous one, which successfully address these limitations. In addition, we also demonstrated the effect of the switching threshold (T_th_) of ICG-NPs and background temperature on USF images, which is an important step towards future *in vivo* studies.

## Results

### Shelf Life Studies in Terms of USF Switching Properties of Three Different Types of ICG-NPs

**Figure 1:**
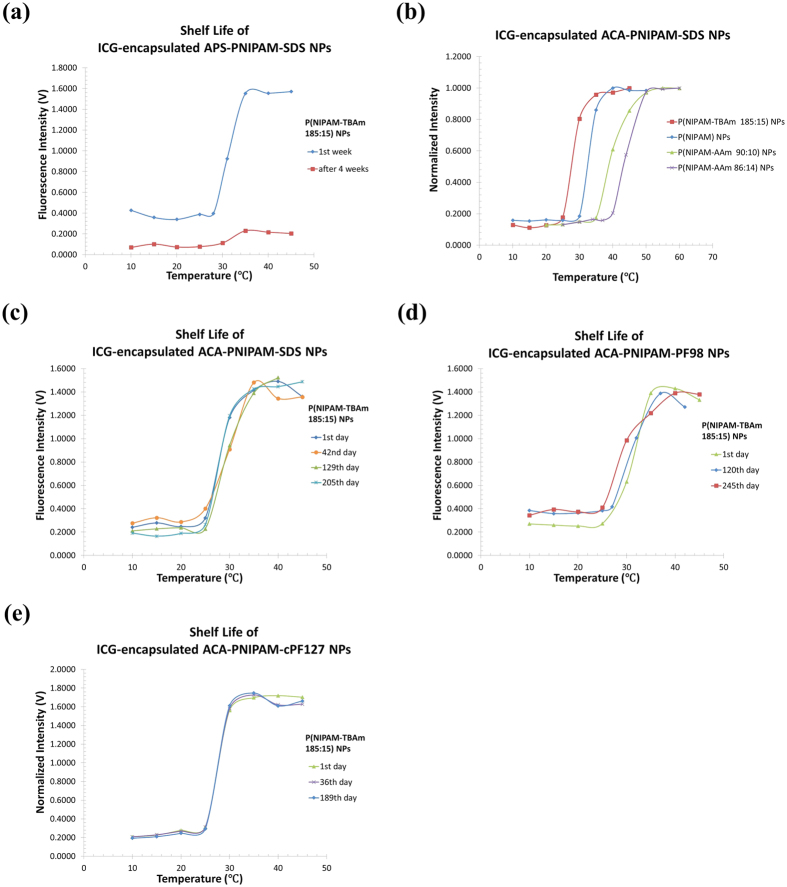
(**a**) Fluorescence intensity of the ICG-encapsulated APS-PNIPAM-SDS NPs nanoparticles as a function of temperature at different days (the 1^st^ day and over 4 weeks) after the synthesis. (**b**) Normalized fluorescence intensity of the four ICG-encapsulated ACA-PNIPAM-SDS NPs with four LCSTs as a function of temperature. (**c**) Fluorescence intensity of the ICG-encapsulated ACA-P(NIPAM-TBAm 185:15)-SDS NPs as a function of temperature at different days (the 1^st^ day, 129^th^ day and 205^th^ day) after the synthesis. (**d**) Fluorescence intensity of the ICG-encapsulated ACA-P(NIPAM-TBAm 185:15)-PF98 NPs as a function of temperature at different days (1^st^ day, 120^th^ day and 245^th^ day) after the synthesis. (**e**) Fluorescence intensity of the ICG-encapsulated ACA-P(NIPAM-TBAm 185:15)-cPF127 NPs as a function of temperature at different days (1^st^ day, 36^th^ day and 189^th^ day) after the synthesis.

The USF mechanism of the contrast agents has been discussed in our previous work^15^,^24^. Briefly, ICG were encapsulated into thermo-sensitive nanoparticles synthesized using thermo-sensitive polymers (PNIPAM). The ICG-NPs exhibit switch-like fluorescence intensity as a function of temperature (see Fig. 1(a–d)). The switching transition between the two states (off and on) is reversible and repeatable^15^. Thus, if a high intensity focused ultrasound (HIFU) can control the tissue temperature in its focal area, we can use ultrasound to switch on the fluorescence emission^15^,^24^,^25^.

In our previous work, we developed a protocol to synthesize ICG-NPs for USF imaging (named as first generation ICG-NPs)^15^. By adjusting thermo-sensitive polymer components and their ratios, the temperature threshold of ICG-NPs can be controlled. For example, the following four ICG-NPs were synthesized and their temperature thresholds were found to be around 28, 31, 37, and 41 °C, respectively: (1) ICG-encapsulated P(NIPAM-TBAm 185:15) NPs, (2) ICG-encapsulated PNIPAM NPs, (3) ICG-encapsulated P(NIPAM-AAm 90:10) NPs, and (4) ICG-encapsulated P(NIPAM-AAm 86:14) NPs^15^. While all these agents show excellent temperature-switching properties, we found that their shelf life is less than 4 weeks (stored at 4 °C). As one example, Fig. 1(a) shows the switching property of ICG-encapsulated P(NIPAM-TBAm 185:15) NPs (measured at 1 and 4 weeks after the sample was synthesized). Clearly, the sample still exhibited an excellent switching property 1 week after the synthesis (with I_ON_/I_OFF_ = ~4.0). However, the agent degraded after 4 weeks because the I_ON_/I_OFF_ was reduced to ~2.0. Moreover, the fluorescence intensity reduced significantly under the same experimental condition. The first-generation ICG-NP adopts ammonium persulfate (APS) as reaction initiator. Meanwhile, it adopts sodium dodecyl sulfate (SDS) as surfactant. Therefore, we also call them ICG-encapsulated APS-initiated SDS-surfactant poly(N-isopropylacrylamide) nanoparticles. For short, we donate them as ICG-encapsulated APS-PNIPAM-SDS NPs. In addition, we found that the ICG-encapsulated APS-PNIPAM-SDS NPs solution appears brown. The brown color is likely due to ICG oxidization during the synthesis because the reaction initiator APS and tetramethylethylenediamine (TEMED) serve as strong oxidizers to decompose alkene structures in ICG. This may be the major factor in its short shelf life in terms of the temperature-switching property^26^.

To overcome this issue, we developed two new synthesis protocols. In one new protocol (synthesized agents are named as second generation ICG-NPs), we replaced the initiator (APS) adopted in the old protocol with a new initiator 4-4′-azobis(4-cyanopentanoic acid) (ACA) to avoid possible oxidization. The ACA works with high reaction efficiency at relatively high temperature (70 °C) with an oil bath. The oil bath makes this synthesis reaction more controllable and avoids secondary reactions before synthesis starts. The ICG-NP solution synthesized from this protocol appears pink. The second-generation ICG-NP adopts 4-4′-azobis(4-cyanopentanoic acid) (ACA) as reaction initiator. Therefore, we also call them ICG-encapsulated ACA-initiated SDS-surfactant poly(N-isopropylacrylamide) nanoparticles. For short, we donate them as ICG-encapsulated ACA-PNIPAM-SDS NPs. Similarly, ICG-encapsulated ACA-PNIPAM-SDS NPs with four different temperature-switching thresholds were synthesized. Figure 1(b) shows their normalized fluorescence intensity as a function of temperature. Like the temperature thresholds of first-generation ICG-NP^15^, the four temperature thresholds are 26, 30, 36, and 40 °C, respectively, which are suitable for both *ex vivo* (such as 26 °C) and *in vivo* (such as 40 °C) USF imaging. More importantly, we found that the shelf life of the second-generation ICG-NP has significantly improved. As one example, Fig. 1(c) shows the temperature-switching properties of the ICG-encapsulated ACA-P(NIPAM-TBAm 185:15)-SDS NPs at three different time points: 1, 129, and 205 days after the sample was synthesized (stored at 4 °C). Clearly, the switching properties remain similar after 205 days (I_ON_/I_OFF_ = 6.0 on the 1^st^ day and I_ON_/I_OFF_ = 5.4 on the 205^th^ day); meanwhile, fluorescence intensity is not reduced, which indicates that this ICG-NP solution is more stable and suitable for long-term storage. Similar results are also found for the other ICG-encapsulated ACA-PNIPAM-SDS NPs (with switching thresholds of 30, 36, and 40 °C; data not shown).

In another new protocol (synthesized agents are named as third generation ICG-NPs), we still used ACA as the initiator (reaction temperature: 70 °C), but replaced the surfactant sodium dodecyl sulfate (SDS) adopted in the previous two protocols with a new surfactant, Pluronic F98 (containing two hydroxyl end-groups) or carboxylized Pluronic F127 (containing two carboxyl end-groups; see Supplementary Information for more details). The ICG-NP solution synthesized from this protocol appears purple. The third-generation ICG-NP adopts ACA as reaction initiator. Meanwhile, it adopts Pluronic F98 or carboxylized Pluronic F127 as new surfactant. Therefore, we also call them ICG-encapsulated ACA-initiated Pluronic-surfactant poly(N-isopropylacrylamide) nanoparticles. For short, we donate them as ICG-encapsulated ACA-PNIPAM-Pluronic NPs: ICG-encapsulated ACA-PNIPAM-PF98 NPs or ICG-encapsulated ACA-PNIPAM-cPF127 NPs. The hydroxyl groups on Pluronic F98 or the carboxyl groups on carboxylized pluronic F127 can be used for conjugation with other targeting moieties such as peptides, proteins or antibodies for the purpose of molecular targeting in the future. The *Methods* section provides details about the two new protocols. Similarly, the third-generation ICG-NPs are also very stable. As an example, Fig. 1(d) shows the temperature-switching properties of the ICG-encapsulated ACA-P(NIPAM-TBAm 185:15)-F98 NPs at three different time points: 1, 120, and 245 days after the sample was synthesized (stored at 4 °C). The switching properties remain similar after 245 days (I_ON_/I_OFF_ = 4.9 on the 1^st^ day and I_ON_/I_OFF_ = 4.1 on the 245^th^ day); meanwhile the fluorescence intensity does not decrease, indicating that this ICG-NP solution is also suitable for long-term storage. Figure 1(e) shows similar results of the ICG-encapsulated ACA-P(NIPAM-TBAm 185:15)-cPF127 NPs at three different time points: 1, 31, and 189 days after the sample was synthesized (stored at 4 °C).

Table 1 summarizes the performance of the three different types of ICG-NPs with (1) laser excitation wavelength λ_ex_ (nm) and the wavelength of the adopted fluorescence emission filter λ_em_ (nm), (2) the fluorescence intensity ratio between on and off states (I_on_/I_off_), (3) the temperature threshold to switch on fluorescence (T_th_), (4) the fluorescence lifetime ratio between on and off states (τ_on_/τ_off_) and the fluorescence lifetime of on states (τ_on_), (5) the temperature transition bandwidth (T_BW_), and (6) their shelf lives. The shelf life data in Table 1 represent the longest time that we could measure after the synthesis of the sample when preparing this paper, but the shelf life could be longer.

### USF Imaging Using the Three Different Types of ICG-Based Contrast Agents

**Figure 2:**
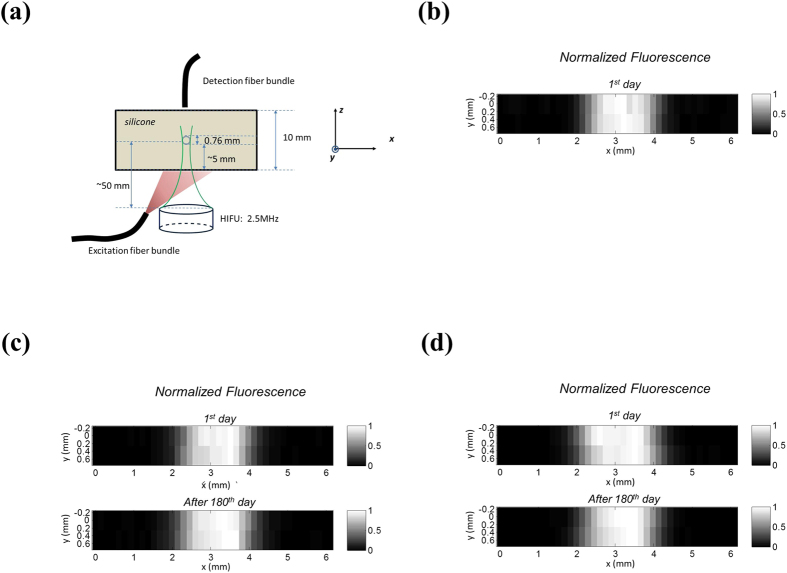
(**a**) The sample configuration, including the silicone phantom, the silicone tube (0.76 mm), the excitation and detection fiber bundle and the HIFU transducers (2.5 MHz). (**b**) USF image of the silicone phantom imbedded with the silicone tube (0.76 mm) using the ICG-encapsulated APS-P(NIPAM-TBAm 185:15)-SDS NPs (LCST = 28 °C) on 1^st^ day after synthesis. (**c**) USF image of the silicone phantom imbedded with the silicone tube (0.76 mm) using ICG-encapsulated ACA-P(NIPAM-TBAm 185:15)-SDS NPs (LCST = 26 °C). The top sub-image used the NPs on 1^st^ day after synthesis, the bottom sub-image used the NPs after 180^th^ day. (**d**) USF image of the silicone phantom imbedded with the silicone tube (0.76 mm) using ICG-encapsulated ACA-P(NIPAM-TBAm 185:15)-PF98 NPs (LCST = 26 °C). The top sub-image used the NPs on 1^st^ day after synthesis, the bottom sub-image used the NPs after 180^th^ day. The USF images were carried out and normalized based on the data processing method in our previous work^25^.

To investigate whether the ICG-NPs can be used for USF imaging 6 months after their synthesis, we carried out USF imaging at different time points after USF contrast agents are synthesized. A tissue-mimic silicone phantom was adopted as a target. Figure 2(a) shows the sample configuration. The thickness of the phantom is 10 mm (along the z-axis) and the width is 40 mm (along the x-axis). A silicone tube (with an inner diameter of 0.76 mm) is embedded in the silicone phantom (along the y-axis) at a depth of ~5 mm to simulate a blood vessel. Titanium dioxide (with a concentration of 0.06 mg/mL) is uniformly dissolved in the silicone phantom to make it a scattering medium (μ_a_ = 0.03; μ_s_′ = 3.5 cm^−1^)^25^,^27^. The *Methods* section describes details of the sample configuration protocol. To image this tube, it was filled, respectively, with the aqueous solution of the three types of ICG-encapsulated P(NIPAM-TBAm 185:15) NPs (i.e., APS-PNIPAM-SDS NPs, ACA-PNIPAM-SDS NPs, ACA-PNIPAM-PF98 NPs). We adopted the USF imaging system that we had used in our previous publication^25^.

Figure 2(b) shows the USF image of the tube on the x-y plane acquired from the ICG-encapsulated APS-P(NIPAM-TBAm 185:15)-SDS NPs (i.e., the first-generation ICG-NPs) right after the agents were synthesized (defined as the 1^st^ day). The HIFU transducer has a 2.5 MHz central frequency (H-108, Sonic Concepts Inc, Bothell, WA, USA). The peak-to-peak driving voltage (V_pp_) from the function generator is 120 mV and is further amplified 50 dB via a power amplifier (325LA, E&I, Rochester, NY, USA) before it is applied to the transducer. The HIFU exposure time is 300 ms. The FWHM of the image is 1.70 mm and the SNR of the image is 109 (SNR is defined as the ratio of the peak USF signal to the standard deviation of the background noise). After 4 weeks, we conducted the same experiment using the same batch agent. We found the quality of the USF image unacceptable (the image is not shown).

Figure 2(c) shows the USF images of the same silicone phantom using the the ICG-encapsulated ACA-P(NIPAM-TBAm 185:15)-SDS NPs (i.e., the second-generation ICG-NPs). The top and bottom images represent, respectively, the USF images acquired on the 1^st^ day and after the 180^th^ day after the agent was synthesized under the same experiment. The same HIFU transducer was used (2.5 MHz). However, the driving voltage from the function generator was slightly reduced (V_pp_ = 90 mV) while the HIFU exposure time remained 300 ms. The FWHMs of the top and bottom USF images are 1.70 and 1.64 mm, respectively, and the corresponding SNRs are 154 and 178.5. Similar results were achieved for the the ICG-encapsulated ACA-P(NIPAM-TBAm 185:15)-PF98 NPs (i.e., the third-generation ICG-NPs) and shown in Fig. 2(d). The experimental parameters remained the same as those for the above two experiments except that the HIFU driving voltage was V_pp_ = 110 mV. The FWHMs of the top and bottom USF images are 1.70 and 1.90 mm, respectively, and the corresponding SNRs are 304 and 292. Figure 2(c,d) indicate that the qualities of both the ICG-encapsulated ACA-P(NIPAM-TBAm 185:15)-SDS NPs and the ICG-encapsulated ACA-P(NIPAM-TBAm 185:15)-PF98 NPs remain high and that both can be used for USF imaging even after 180 days. Table 2 provides a summary of USF imaging performance of the three ICG-NPs.

### The Effect of the Temperature-Switching Threshold and the Background Temperature on USF Imaging

**Figure 3:**
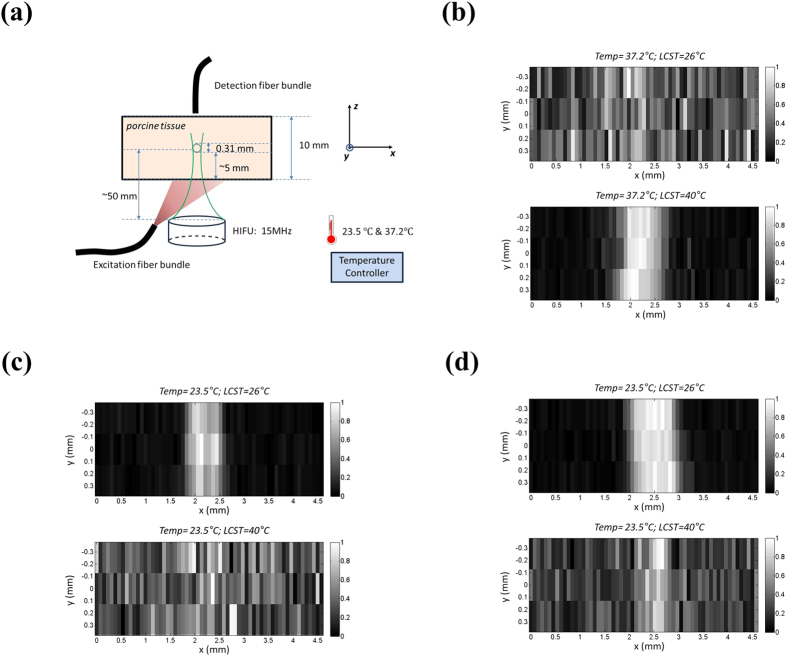
(**a**) The sample configuration, including the porcine tissue phantom, the silicone tube (0.31 mm), the excitation and detection fiber bundle, the HIFU transducers (15 MHz) as well as the temperature controller. The whole sample was merged into a big water tank and the sample temperature was controlled by water bath with the temperature controller. (**b**) USF image of the sample with experimental temperature at 37.2 °C. ICG-encapsulated ACA-PNIPAM-SDS NPs were used as the contrast agents; the top sub-image shows the USF image using ICG-encapsulated ACA-P(NIPAM-TBAm 185:15)-SDS NPs with LCST = 26 °C, the bottom sub-image shows the USF image using ICG-encapsulated ACA-P(NIPAM-AAm 85:15)-SDS NPs with LCST = 40 °C. The HIFU transducer driving voltage is 500 mV with a duration of 200 ms (single burst). (**c**) USF image of the same experimental condition as (**b**), except that the experimental temperature is 23.5 °C and the HIFU transducer driving voltage is 350 mV with a duration of 200 ms (single burst). (**d**) USF image of the same experimental condition as (**b**), except that the experimental temperature is 23.5 °C and the HIFU transducer driving voltage is 500 mV with a duration of 200 ms (single burst). The USF images were carried out and normalized based on the data processing method in our previous work^25^.

To investigate the effect of the temperature-switching threshold (T_th_) and the background temperature (T_BG_) on USF imaging, we conducted the following experiments. First, we synthesized two ICG-encapsulated ACA-PNIPAM-SDS NPs contrast agents with LCST = 26 °C (i.e., ICG-encapsulated ACA-P(NIPAM-TBAm 185:15)-SDS NPs) and 40 °C (i.e., ICG-encapsulated ACA-P(NIPAM-AAm 85:15)-SDS NPs). Second, we made a tissue phantom by inserting a small silicone tube (with an inner diameter of 0.31 mm) into a piece of porcine muscle tissue (with a thickness of 10 mm). Figure 3(a) shows the sample configuration. Third, the two ICG-NP solutions were injected, respectively, into the tube for USF imaging. Because the inner diameter of the tube (0.31 mm) is smaller than the previous one (0.76 mm), a relatively higher spatial resolution is preferable. Therefore, we replaced the previous 2.5-MHz HIFU transducer with a 15-MHz HIFU transducer (H-202, Sonic Concepts Inc, Bothell, WA, USA), thus reducing the lateral acoustic focal size from ~420 to ~100 microns. In addition, we used a temperature controller (with a heater and a temperature detection probe) to control the temperature of the water bath in which the phantom was submerged. Also, we used a magnetic stirrer (11-100-16S, Fisher Scientific, USA) along with a long magnetic bar to stir the whole water in the tank to stabilize the water temperature. Other parts of the USF system remained the same. Finally, we imaged the tube with the USF system at two different background temperatures by controlling the temperature of the water bath: 23.5 and 37.2 °C. The former and the latter simulate *ex vivo* and *in vivo* background temperatures, respectively.

Figure 3(b) shows the USF images acquired at the background temperature of 37.2 °C from the two ICG-encapsulated ACA-PNIPAM-SDS NPs with LCST = 26 °C (top) and LCST = 40 °C (bottom). The driving voltage of the 15-MHz HIFU transducer from the function generator was V_pp_ = 500 mV, while the exposure time was 200 ms. The USF image clearly shows the tube filled with ICG-NP agent with LCST = 40 °C (the bottom figure). The FWHM of the image is 0.868 ± 0.074 mm and the SNR is 37.70 ± 2.69. However, the tube cannot visualized from the USF image when it was filled with ICG-NP agent with LCST = 26 °C (the top figure). We expected this result because the 37.2 °C background temperature may already completely switch on the ICG-NPs with LCST = 26 °C (i.e., the background temperature is above the temperature-switching threshold). Therefore, ICG-NPs do not respond further to an ultrasound-induced temperature increase in tissue. On the other hand, the 37.2 °C background temperature is not high enough to switch on the ICG-NPs with LCST = 40 °C (i.e., the background temperature is below the temperature-switching threshold) and the ICG-NPs remain intact. Accordingly, when ultrasound induces the tissue temperature above the threshold in its focal region, ICG-NPs are switched on and therefore the tube can be clearly imaged via USF. This result provides an excellent model for future *in vivo* studies because the background temperature of a living body (either vertebrate animals or human) is around 37 °C.

Similarly, we performed another experiment at room temperature to simulate an *ex vivo* scenario. The background temperature of the water bath was 23.5 °C. In this experiment the HIFU driving voltage from the function generator was V_PP_ = 350 mV and the exposure time remained 200 ms. Figure 3(c) shows the USF images of the tube filled with ICG-NPs with LCST = 26 °C (top) and LCST = 40 °C (bottom). The tube is clearly imaged when filled with agents with low LCST (26 °C). The FWHM is 0.701 ± 0.012 mm and the SNR is 34.04 ± 2.02. However, the tube filled with agents with high LCST (40 °C) cannot be visualized using current parameters. This result indicates that the final temperature in the ultrasound focal region (the sum of the background temperature and the ultrasound-induced temperature) is high enough to switch on a significant number of ICG-NPs that have a low temperature-switching threshold (26 °C). However, the final temperature is still low and unable to switch on a significant number of ICG-NPs that have a relatively high temperature-switching threshold (40 °C). To switch on these high LCST ICG-NPs, a higher focal temperature is needed. To validate this idea, we increased the HIFU driving voltage from 350 mV to 500 mV while the exposure time remained 200 ms. We expected that the final temperature in the focal region should be increased. Figure 3(d) shows the results. As the bottom image shows, the tube filled with high LCST agents can be roughly visualized (compared with the bottom image in Fig. 3(c)), although the image is still noisy. This indicates that ultrasound has switched on some number of ICG-NPs, which validates the assumption. Furthermore, the SNR of the USF image acquired from the low LCST agents (the top image) is increased to 47.66 ± 1.41 (compared with 34.04 ± 2.02 of the one in Fig. 3(c)), indicating that more ICG-NPs were switched on compared with those in Fig. 3(c) because HIFU induced higher temperature. In addition, the FWHM of this image is 0.927 ± 0.023 mm, wider than the one in Fig. 3(c) (0.701 ± 0.012 mm). This is understandable because the higher the temperature that ultrasound induces, the larger the thermal volume is, therefore the lower the spatial resolution is. Table 3 summarizes the details of this study.

## Discussion

High-resolution USF imaging in centimeter-deep tissues is a new technology, highly dependent on the contrast agents adopted^15^,^16^,^24^. The ICG-NPs are one family of excellent USF contrast agents because they have reasonable I_ON_/I_OFF_, NIR excitation and emission spectra, narrow temperature transition bandwidths, and controllable temperature-switching thresholds. In this work, we addressed three additional challenges for this family of contrast agents, further reducing the application barriers of this technology.

First, we developed two new protocols that significantly increased the stability of USF switching properties. The shelf life of ICG-NP was increased from less than one month to longer than 6 months. We expect that the shelf life of both the two new ICG-NPs (i.e., ICG-encapsulated ACA-PNIPAM-SDS NPs and ICG-encapsulated ACA-PNIPAM-Pluronic NPs) will be even longer than one year because we do not see the performance degradation in this work. Long-term stability of USF contrast agents is highly desirable for some experiments. For example, when conducting comparative longitudinal studies such as monitoring disease development and treatment evaluation over a long period (usually > 6 months), adopting USF contrast agents synthesized from the same batch can avoid those unwanted variations that originate from the different batches of production. Meanwhile, fast degradation will make it challenging or sometimes even impossible to maintain contrast agents with same parameters and stable properties. In addition, stable contrast agents can save a large amount of effort (such as time and cost) by avoiding having to repetitively make new batch agents to replace quickly degraded agents. This is the key to reducing the cost of agent and further increasing its commercialization potential. Finally, a long-term stable USF contrast is necessary for applications that use USF imaging to monitor implanted medical device, in which USF contrast agents are sealed into the medical device for long-term use (up to years), making USF performance stability extremely important.

Second, the success of replacing the original surfactant (SDS) of our previous ICG-NPs with Pluronic F98 or carboxylated Pluronic F127 provides a great opportunity to functionalize ICG-NPs in future. Molecular imaging has achieved significant progress in past years. Functionalizing the surface of contrast agents is an important future goal for conducting USF molecular imaging in deep tissue with high resolution. Unlike the sodium sulfate (SDS) end-groups, the two hydroxyl end-groups (F98) or the two carboxyl end-groups (carboxylated F127) are exposed to the surrounding hydrophilic aqueous solution and show potential to react with other molecules containing amine or carboxyl groups. Thus, these function groups could possibly be conjugated with targeting moieties such as antibodies and ligands to target specific molecules or proteins.

In addition, we demonstrated for the first time the effect of the temperature-switching threshold (T_th_) and the background temperature (T_BG_) on USF imaging. For ICG-NP based contrast agents, the results indicate that USF imaging can be successfully or efficiently conducted when T_th_ is controlled slightly above T_BG_ (the bottom figure in Fig. 3(b); the top figures in Fig. 3(c,d)). In contrast, when T_th_ is much higher than T_BG_, USF imaging can fail (the bottom figures in Fig. 3(c)) or need stronger energy (the bottom figures in Fig. 3(d)). Finally, when T_th_ is well below T_BG_, USF imaging can fail (the top figure in Fig. 3(b)). Based on these results, a very important indication for future *in vivo* studies is that the T_th_ of the ICG-NP based contrast agents should be controlled ~38–40 °C, which is slightly above the body temperature (37 °C). We will investigate this in future research.

In summary, the development of new USF contrast agents is essential for the development of the USF technique and its applications. This work plays an important role for future USF *in vivo* study.

## Conclusions

In conclusion, two new protocols of ICG-P(NIPAM) nanoparticles were developed for USF contrast agents with long-term stability, adjustable LCST, and functionalization. The shelf life of these new generation ICG-NPs developed in this study is much longer (>6 months) than that of the first generation (<1 month), and their quality remains good as of this writing. We have also synthesized the new ICG-NPs with conjugation-functional groups (hydroxyl or carboxyl), which can be used for molecular imaging in the future if they are attached with a targeting moiety. Finally, we investigated the effect of the temperature-switching threshold of the agent and the background temperature on USF imaging. Maintaining the agents’ temperature threshold slightly above the background temperature is necessary for conducting USF imaging successfully and efficiently. Therefore, for *in vivo* USF imaging, the temperature threshold of the contrast agents should be controlled around 38–40 °C, which is slightly above the body temperature of ~37 °C. Overall, this work makes a firm step for future research on USF imaging.

## Methods

### Chemical Materials

N-isopropylacrylamide (NIPAM), acrylamide (AAm), ammonium persulfate (APS), 4-4′-Azobis(4-cyanopentanoic acid) (ACA), sodium dodecyl sulfate (SDS), N,N,N′,N′-tetramethyl ethylene diamine (TEMED), N,N’-methylenebisacrylamide (BIS), N-tert-butylacrylamide (TBAM), succinic anhydride, Pluronic F127, dimethyl sulfoxide (DMSO) and indocyanine green (ICG) were purchased from Sigma-Aldrich (St. Louis, MO, USA). Pluronic F98 Pastille was purchased from BASF Corporation (Vandalia, IL, USA). All chemicals are used as purchased without further purification.

### Synthesis Protocols

Protocol A (i.e., the ICG-encapsulated APS-PNIPAM-SDS NPs): We used the same protocol in terms of our previous work^15^. Basically, 0.6822 g NIPAM, 0.0131 g BIS and 0.0219 g SDS were dissolved with 50 mL de-ionized water in a 250 mL Schlenk tube, followed by nitrogen bubble purging for 10 minutes. AAm or TBAm was added by different ratio with NIPAM (i.e., NIPAM: TBAm = 185:15; pure NIPAM; NIPAM: AAm = 90:10; NIPAM: AAm = 86:14) to adjust the T_th_ (i.e., LCST) of NPs. 0.0034 g ICG, 0.039 g APS, and 51 μL TEMED were added into the tube, and then the tube was vacuumed and nitrogen-purged alternately for three times to give a nitrogen-protected environment for reaction. The reaction was carried out at room temperature for 4 hours, and stopped by exposing to air by loosening the valve. The sample was dialyzed in 3-liter deionized water using a 10-kDa molecular weight cut-off membrane for 3 days to remove extra unreacted materials. Protocol B (i.e., the ICG-encapsulated ACA-PNIPAM-SDS NPs): We made the following changes based on Protocol A. Instead of APS, ACA was employed as the initiator (0.070 g). The reaction was carried out at 70 °C overnight in the absence of TEMED (It may react with ICG and change the color from green to brown, as in Protocol A). In addition, the quantity of monomer NIPAM is doubled. The synthesized ICG-NP solution appears pink. Protocol C (i.e., the ICG-encapsulated ACA-PNIPAM-Pluronic NPs): Instead of SDS in Protocol A and B, a new surfactant Pluronic F98 (0.329 g) or carboxylated Pluronic F127 (0.3164 g) is used. The reaction was carried out at 70 °C overnight with the initiator ACA. The quantity of monomer NIPAM is doubled. The synthesized ICG-NP solution appears purple.

### Synthesis of Carboxylized Pluronic F127

Pluronic F127 (8.5 g, 0.68 mmol) was dissolved in DMSO in a 250 mL 3-neck flask. The succinic anhydride (0.34 g, 3.4 mmol) in 5-fold excess was then added dropwise into the Pluronic F127/DMSO solution. The mixture was stirred at 60 °C for 24 hours under nitrogen atmosphere. The final product was collected after dialysis in distilled water for 3 days and lyophilization for 2 days. The chemical structure of synthesized carboxylized Pluronic F127 was confirmed by H^1^ NMR (D_2_O, 300 MHz) and ATR-FTIR. (See Supplementary Information.)

### Sample Configuration Protocol of Silicone Phantoms and Tissue Phantoms

The silicone kit was purchased from Factor II Inc. (VST-50: VerSilTal Silicone Elastomer). The kit includes two major components: silicone elastomer and catalyst. First, 1.33 mg titanium dioxide (TiO_2_) was dissolved in 2 mL silicone catalyst. Then, it was mixed with 20 mL silicone elastomer. TiO_2_ functions light scatters in the silicone phantom and the estimated absorption coefficient μ_a_ = 0.03 and reduced scattering coefficient μ_s_′ = 3.5 cm^−1^^25^,^27^. The mixture was poured into a small plastic container and a silicone tube was inserted through the wall of container at an appropriate depth. The container was placed into a vacuum to remove small bubbles inside the silicone. Then, the silicone phantom was solidified at room temperature (for about 12 hours). Finally, the container was peeled off and the silicone phantom was ready to use. The porcine muscle tissue sample was prepared as follows. A silicone tube was carefully inserted into a piece of porcine muscle tissue with a thickness of 10 mm. Both the top and bottom sides of the porcine tissue were covered with ultrasound jell (01–08, AQUASONIC^®^ 100, Parker Laboratories Inc., Fairfield, New Jersey, USA) to maintain appropriate ultrasound coupling. A piece of transparent parafilm (PM-992, BEMIS Company Inc. Neenah, WI, USA) was used to wrap the tissue and separate the sample from water to enable the tissue to maintain a natural status for a long time during experiments without drying from air exposure or degrading from water exposure.

### Measuring Fluorescence Intensity as a Function of Temperature

We adopted the same system as in our previous study for measuring the fluorescence intensity of the ICG-NPs as a function of temperature^24^. Briefly, a sub-nanosecond pulsed and nitrogen-pumped dye-laser (peak wavelength: 775 nm) was filtered by a band-pass filter (749/789 nm) and used as the excitation source. An optical alignment system was adopted to collimate the fluorescence light and two 830-nm long-pass filters were used to block the excitation light. A photomultiplier tube (PMT) was adopted as the detector and a multichannel oscilloscope for data acquisition. 3 mL ICG-NP solutions were prepared in a quartz cuvette submerged in a water bath via a small transparent glass container. The water temperature is controlled via a temperature controller, a heater, and a temperature detection probe. See Supplementary Information for more details.

### Fluorescence Lifetime Measurement

Fluorescence lifetime of ICG-NPs was measured via a gated ICCD camera system synchronized with a pico-sec laser in a customized inverted microscope system. The width of the impulse response function (IRF) of the ICCD camera system with the pico-second pulsed laser was ~250 ps. The measured ICG fluorescence signal was then de-convolved with the IRF to calculate the fluorescence lifetime of the ICG-NPs. To study whether the fluorescence lifetime of the ICG-NPs changes with temperature, the experiments were conducted both at room temperature (<Tth, ~25 °C) and high temperature (>Tth, ~50 °C). See Supplementary Information for more details. Data of the ICG-NPs fluorescence lifetime is shown in Table 1.

### USF Imaging System

The imaging system is similar to the one we adopted in our previous studies^25^. Briefly, the intensity of the excitation laser (808 nm) was modulated at 1 kHz frequency using a function generator. A band-pass excitation filter (785/62 nm) was positioned in front of the laser to block any unknown emission from the laser with wavelengths outside the pass-band. The 1-kHz modulated fluorescence emission was filtered through an optical collimation and emission filter system (with three 830-nm long-pass interference filters and two 830-nm absorption filters) to maximally block the excitation light and pass the fluorescence photons. A cooled photomultiplier tube (PMT) was used as a detector and its output signal was further amplified via a low noise preamplifier. A lock-in amplifier (LIA) was used to detect the amplitude variation of the 1-kHz fluorescence signal. The sensitivity of the pre-amplifier was tuned to 50 nA/V, and the lock-in time constant was 300 ms. The LIA output was acquired using a data acquisition card. A high intensity focused ultrasound (HIFU) transducer was adopted for USF imaging. The HIFU was focused inside the sample to heat it at HIFU focal zone and then switch on the ICG-NP based USF contrast agents. When HIFU was turned on, the amplitude of the 1-kHz fluorescence emission would increase. This ultrasound-induced amplitude change can be detected by the LIA, which was recorded as USF signal strength at that location. A motorized translation stage was used to scan the sample on the x-y plane to acquire a USF image. A pulse delay generator (PDG) was used as a master trigger to synchronize all subsystems and signals. See Supplementary Information for more details.

## Additional Information

**How to cite this article**: Yu, S. *et al.* New generation ICG-based contrast agents for ultrasound-switchable fluorescence imaging. *Sci. Rep.* **6**, 35942; doi: 10.1038/srep35942 (2016).

## Supplementary Material

Supplementary Information

## Figures and Tables

**Table 1:** Summary of ICG-NP’s USF performance.

	λ_ex_/λ_em_ (nm)	I_on_/I_off_	T_th_ or LCST (°C)	τ_on_/τ_off_ τ_on_ (ns)	T_bw_ (°C)	Shelf-life
ICG-encapsulated APS-PNIPAM-SDS NPs	775 nm/2 × 830 nm LP	~4	~28 °C*^1^	~3.1 ~ 0.50 ns	~5 °C	<30 days
ICG-encapsulated ACA-PNIPAM-SDS NPs	775 nm/2 × 830 nm LP	~6	~26, 30, 36, 40 °C	~3.1, 2.6, 2.6, 2.4 ~ 0.45, 0.49, 0.40, 0.40 ns	~5–8 °C	>180 days*^6^ for all LCSTs
ICG-encapsulated ACA-PNIPAM-PF98 NPs *^4^	775 nm/2 × 830 nm LP	~5	~26 °C*^2^	~2.4 ~ 0.48 ns	~8 °C	>180 days*^7^
ICG-encapsulated ACA-PNIPAM-cPF127 NPs *^5^	775 nm/2 × 830 nm LP	~5	~26 °C*^3^	~3.0 ~ 0.57 ns	~5 °C	>180 day*^8^

**Table 2:** Summary of ICG-NP’s USF performance.

	HIFU central frequency & Power	Target size & Imaging Depth	Scattering Medium	SNR*^11^	FWHM*^12^ (mm)	Shelf-life
ICG-encapsulated APS-PNIPAM-SDS NPs(LCST = 28 °C)	2.5 MHz 120 mV	760 um & 10 mm	TiO2 Silicone Phantom	~109	~1.70	<30 days
ICG-encapsulated ACA-PNIPAM-SDS NPs(LCST = 26 °C)	2.5 MHz 90 mV	760 um & 10 mm	TiO2 Silicone Phantom	~166	~1.67	>180 days*^9^
ICG-encapsulated ACA-PNIPAM-F98 NPs(LCST = 26 °C)	2.5 MHz 110 mV	760 um & 10 mm	TiO2 Silicone Phantom	~298	~1.80	>180 days*^10^

**Table 3:** Summary of ICG-NP’s USF performance.

	HIFU central frequency & Power	Target size & Imaging Depth	Scattering Medium	SNR*^11^	FWHM*^12^ (mm)	Sample Temperature (°C)
ICG-encapsulated ACA-PNIPAM-SDS NPs(LCST = 26 °C)	15 MHz 350 mV	310 um & 10 mm	Porcine Tissue Phantom	~34.0	~0.70	23.5
ICG-encapsulated ACA-PNIPAM-SDS NPs(LCST = 26 °C)	15 MHz 500 mV	310 um & 10 mm	Porcine Tissue Phantom	~47.6	~0.93	23.5
ICG-encapsulated ACA-PNIPAM-SDS NPs(LCST = 40 °C)	15 MHz 500 mV	310 um & 10 mm	Porcine Tissue Phantom	~37.7	~0.87	37.2

*1,2,3: The LCST of both ICG-encapsulated APS-PNIPAM-SDS NPs and ACA-PNIPAM-Pluronic NPs could be adjusted by change the molecular ratio of monomer NIPAM, TBAm, AAm in synthesis of nanoparticles. Typically, for ICG-encapsulated APS-PNIPAM-SDS NPs, LCST could be adjusted to 28 °C (NIPAM-TBAm 185:15), 31 °C (NIPAM 100%), 37 °C (NIPAM-AAm 90:10), and 41 °C (NIPAM-AAm 86:14) according to our previous paper^15^. For ICG-encapsulated ACA-PNIPAM-SDS NPs, LCST could be adjusted to 26 °C (NIPAM-TBAm 185:15), 30 °C (NIPAM 100%), 36 °C (NIPAM-AAm 90:10), 40 °C (NIPAM-AAm 85:15), similar to for the ICG-encapsulated ACA-PNIPAM-Pluronic NPs.

*4,5: There are two types of ICG-encapsulated ACA-PNIPAM-Pluronic NPs: one adopts surfactant pluronic F98 (containing two hydroxyl groups at its hydrophilic ends) and the other adopts surfactant carboxylized pluronic F127 (containing two carboxyl groups at its hydrophilic ends).

*6,7,8,9,10: The data about the shelf life in Tables 1 and 2 represents the longest time that we can measure after the sample synthesis when preparing this paper and their shelf life may be longer than that.

*11,12: SNR and FWHM refers to the mean of signal-to-noise-ratio and the mean of full-width-half-maximum in each USF scan correspondingly.

## References

[b1] Ntziachristos V., Bremer C. & Weissleder R. Fluorescence imaging with near-infrared light: new technological advances that enable *in vivo* molecular imaging. Eur radiol 13, 195–208 (2003). 12541130 10.1007/s00330-002-1524-x

[b2] Frangioni J. V. *In vivo* near-infrared fluorescence imaging. Curr opin chem biol 7, 626–634 (2003). 14580568 10.1016/j.cbpa.2003.08.007

[b3] Andresen V. *et al.* Infrared multiphoton microscopy: subcellular-resolved deep tissue imaging. Curr opin biotech 20, 54–62 (2009). 19324541 10.1016/j.copbio.2009.02.008

[b4] Corlu A. *et al.* Three-dimensional *in vivo* fluorescence diffuse optical tomography of breast cancer in humans. Opt express 15, 6696–6716 (2007). 19546980 10.1364/oe.15.006696

[b5] Culver J., Akers W. & Achilefu S. Multimodality molecular imaging with combined optical and SPECT/PET modalities. J nucl med 49, 169–172 (2008). 18199608 10.2967/jnumed.107.043331

[b6] Yuan B. & Zhu Q. Separately reconstructing the structural and functional parameters of a fluorescent inclusion embedded in a turbid medium. Opt express 14, 7172–7187 (2006). 18160970 10.1364/oe.14.007172 PMC2153462

[b7] Xu C. T. *et al.* High-resolution fluorescence diffuse optical tomography developed with nonlinear upconverting nanoparticles. ACS nano 6, 4788–4795 (2012). 22568960 10.1021/nn3015807

[b8] Morimoto S. [*In-vivo* imaging of tumors with protease activated near-infrared fluorescent probes]. Tanpakushitsu kakusan koso. Protein, nucleic acid, enzyme 52, 1774–1775 (2007). 18051420

[b9] Hoffman R. M. Green fluorescent protein imaging of tumour growth, metastasis, and angiogenesis in mouse models. Lancet oncol 3, 546–556 (2002). 12217792 10.1016/s1470-2045(02)00848-3

[b10] Si K., Fiolka R. & Cui M. Fluorescence imaging beyond the ballistic regime by ultrasound-pulse-guided digital phase conjugation. Nat photonics 6, 657–661 (2012). 23241552 10.1038/nphoton.2012.205 PMC3521493

[b11] Si K., Fiolka R. & Cui M. Breaking the spatial resolution barrier via iterative sound-light interaction in deep tissue microscopy. Sci rep 2 (2012). 10.1038/srep00748 PMC3475990 23087813

[b12] Wang Y. M., Judkewitz B., DiMarzio C. A. & Yang C. Deep-tissue focal fluorescence imaging with digitally time-reversed ultrasound-encoded light. Nat commun 3, 928 (2012). 22735456 10.1038/ncomms1925 PMC3621452

[b13] Lai P., Suzuki Y., Xu X. & Wang L. V. Focused fluorescence excitation with time-reversed ultrasonically encoded light and imaging in thick scattering media. Laser phys lett 10, 075604 (2013). 10.1088/1612-2011/10/7/075604 PMC3900304 24465244

[b14] Judkewitz B., Wang Y. M., Horstmeyer R., Mathy A. & Yang C. Speckle-scale focusing in the diffusive regime with time reversal of variance-encoded light (TROVE). Nat photonics 7, 300–305 (2013). 23814605 10.1038/nphoton.2013.31 PMC3692396

[b15] Pei Y. *et al.* High resolution imaging beyond the acoustic diffraction limit in deep tissue via ultrasound-switchable NIR fluorescence. Sci rep 4 (2014). 10.1038/srep04690 PMC4003820 24732947

[b16] Yuan B., Uchiyama S., Liu Y., Nguyen K. T. & Alexandrakis G. High-resolution imaging in a deep turbid medium based on an ultrasound-switchable fluorescence technique. Appl phys lett 101, 033703 (2012). 10.1063/1.4737211 PMC3411561 22893732

[b17] Lin Y., Kwong T. C., Bolisay L. & Gulsen G. Temperature-modulated fluorescence tomography based on both concentration and lifetime contrast. J biomed opt 17, 0560071–0560074 (2012). 10.1117/1.JBO.17.5.056007 PMC3381013 22612130

[b18] Lin Y., Bolisay L., Ghijsen M., Kwong T. C. & Gulsen G. Temperature-modulated fluorescence tomography in a turbid media. Appl phys lett 100, 073702 (2012). 10.1063/1.3681378 PMC3292592 22393266

[b19] Liu Y., Feshitan J. A., Wei M.-Y., Borden M. A. & Yuan B. Ultrasound-modulated fluorescence based on fluorescent microbubbles. J biomed opt 19, 085005–085005 (2014). 25104407 10.1117/1.JBO.19.8.085005 PMC4407672

[b20] Huynh N. T., Hayes-Gill B. R., Zhang F. & Morgan S. P. Ultrasound modulated imaging of luminescence generated within a scattering medium. J biomed opt 18, 020505–020505 (2013). 10.1117/1.JBO.18.2.020505 23386195

[b21] Xu X., Liu H. & Wang L. V. Time-reversed ultrasonically encoded optical focusing into scattering media. Nat photonics 5, 154–157 (2011). 21532925 10.1038/nphoton.2010.306 PMC3083021

[b22] Wang L. V. Multiscale photoacoustic microscopy and computed tomography. Nat photonics 3, 503–509 (2009). 20161535 10.1038/nphoton.2009.157 PMC2802217

[b23] Razansky D. *et al.* Multispectral opto-acoustic tomography of deep-seated fluorescent proteins *in vivo*. Nat Photonics 3, 412–417 (2009).

[b24] Cheng B. *et al.* Development of Ultrasound-Switchable Fluorescence Imaging Contrast Agents Based on Thermosensitive Polymers and Nanoparticles. Selected Topics in Quantum Electronics, IEEE Journal of 20, 67–80 (2014). 10.1109/JSTQE.2013.2280997 PMC4454428 26052192

[b25] Cheng B. *et al.* Centimeter-deep tissue fluorescence microscopic imaging with high signal-to-noise ratio and picomole sensitivity. arXiv preprint arXiv : *1510.02112* (2015).

[b26] Engel E. *et al.* Light-induced decomposition of indocyanine green. Invest ophth vis sci 49, 1777–1783 (2008). 10.1167/iovs.07-0911 18436812

[b27] Ayers F., Grant A., Kuo D., Cuccia D. J. & Durkin A. J. In Biomedical Optics (BiOS) 687007–687009 (International Society for Optics and Photonics) (2008).

