# Supplementary material for: New generation ICG-based contrast agents for ultrasound-switchable fluorescence imaging
Source: Sci Rep. 2016 Oct 24;6:35942. doi: 10.1038/srep35942 (PMC5075910; doi:10.1038/srep35942)
Supplement: Supplementary Information [file srep35942-s1.pdf]

Supplementary Information

New generation ICG-based contrast agents for ultrasound-switchable fluorescence imaging

Shuai Yu,[1,2] Bingbing Cheng,[1,2] Tingfeng Yao,[1,2] Cancan Xu,[2,3] Kytai T. Nguyen,[2,3] Yi Hong,[2,3] and Baohong Yuan[1,2*]

[1]*Ultrasound and Optical Imaging Laboratory, Department of Bioengineering, The University of Texas at Arlington, Arlington, TX 76019, USA*

[2]*Joint Biomedical Engineering Program, The University of Texas at Arlington and The University of Texas Southwestern Medical Center at Dallas, TX 75390, USA*

[3]*Department of Bioengineering, The University of Texas at Arlington, Arlington, TX 76019, USA*

System and Instrumentations

Measuring fluorescence intensity as a function of temperature. The system adopted in this study was the same as the one we used in our previous study. [1] Briefly, a sub-nanosecond nitrogen-pumped dye-laser (OL-4300, dye laser: OL-401, both from Optical Building Blocks Corporation, Edison, NJ, USA) was adopted as excitation source. An optical alignment system, a photomultiplier tube (PMT, H10721-20, Hamamatsu, Japan), an amplifier (C5594, Hamamatsu, Japan), and a multichannel GHz oscilloscope (DPO 7254, Tektronix, Beaverton, OR, USA) were adopted as the detector. The excitation laser beam and the detection system formed a 90-degree angle. The ICG-NP sample (~3 mL) was placed in a quartz cuvette (DLC-23-Q-10, White Bear Photonics, LLC, White Bear Lake, MN, USA) at the cross of the laser beam and the detection alignment line. The cuvette filled with the ICG-NP sample was submerged in a transparent glass water tank. The temperature of the water bath was controlled via a temperature controller (PTC10, OMEGA Engineering, Inc., Stamford, CT, USA) coupled with a heater (EMH-060-120V) and a temperature probe (TRP-T). The laser dye (OD 775 Optical Building Blocks Corporation, Edison, NJ) was appropriately selected so that the laser output wavelength was tuned at 775 nm. To further clear up the excitation wavelength, one band pass filter (FF01-769/41, Semrock Inc., Rochester,

NY, USA) was used as an excitation filter. Two long pass filters (BLP01-830R, Semrock Inc., Rochester, NY, USA) were used as the emission filters.

Fluorescence lifetime measurement. Fluorescence lifetime of ICG-NPs was measured via a gated ICCD camera system (Picostar HR, LaVision, Goettingen, Germany) synchronized with a pico-second pulsed laser (SC-450, Fianium Inc., Eugene, OR, USA) in a customized inverted microscope system (Eclipse Ti-U, Nikon), which provides higher accuracy in fluorescence lifetime measurement than our previous fluorescence intensity and lifetime measurement (FILM) system.[1] The width of the impulse response function (IRF) of the ICCD camera system with the pico-second pulsed laser was ~250 ps. The measured ICG fluorescence signal was then de-convolved with the IRF to calculate the fluorescence lifetime of the ICG-NPs. To study whether the fluorescence lifetime of the ICG-NPs changes with temperature, the experiments were conducted both at room temperature ($<T_{th}$, ~25 °C) and high temperature ($>T_{th}$, ~50 °C).

USF imaging system. The USF imaging system was described in our previous studies. [2,3] Briefly, a laser (808 nm, MGL-II-808-2W, Dragon lasers, JL, China) was modulated by a function generator (FG, 33220A, Agilent, Santa Clara, CA, USA) at 1 kHz frequency and used as an excitation source. A band pass filter (FF01-785/62-25, Semrock Inc., Rochester, NY, USA) was used as the excitation filter. To minimize laser leakage, five emission filters were adopted: three long-pass filters (BLP01-830R, Semrock Inc., Rochester, NY, USA) and two absorptive filters (FSRRG830, Newport Corporation, Irvine, CA, USA). The collected fluorescence was refocused on a cooled photomultiplier tube (PMT; H7422-20 driven by a high-voltage source C8137-02, Hamamatsu, Japan). The 1-kHz fluorescence signal from the PMT was further amplified by a low-noise current preamplifier (SR570, Stanford Research Systems, Sunnyvale, CA, USA) and was then input into a lock-in amplifier (LIA; SR830, Stanford Research Systems, Sunnyvale, CA, USA). A synchronized signal from the function generator was used as a reference for the lock-in amplifier. The output of the lock-in amplifier was acquired via a data acquisition card (BNC-2110, National Instruments, Austin, TX, USA). The acquired data were further processed to form USF images. The high intensity focused ultrasound transducers (HIFUs) (2.5 MHz, H-108; 15-MHz, H-202; Sonic Concepts Inc., Bothell, Washington, USA) were adopted for USF imaging. They were driven by gated sinusoidal waves generated by another function generator (33220A, Agilent, Santa Clara,

CA, USA) and further amplified by a power amplifier (325LA, E&I, Rochester, NY, USA). The HIFU signal was focused in the sample to heat its focal zone and thus to switch on ICG molecules, which further induced the amplitude change of the 1-kHz fluorescence signal. A motorized translation stage (Velmex Inc. Bloomfield, NY, USA) was used to scan the sample. A pulse delay generator (PDG; P400, Highland Technology Inc., San Francisco, CA, USA) was used as a master trigger to synchronize the different systems.

Characterization of USF contrast agents

The diameter of ICG-NPs was measured by dynamic light scattering (DLS) at room temperature (~25 °C). 50uL of the sample was diluted with 2.95 mL deionized water and then measured with Nanotrac 150 (Microtrac, Inc., Nikkiso, San Diego, CA, USA). The size of ICG-NPs varies from different synthesis protocols and different batches. Typically, the ICG-encapsulated ACA-PNIPAM-SDS NPs have a size range of 250-350 nm; the ICG-encapsulated ACA-PNIPAM-PF98 NPs have a size range of 150-200 nm; the ICG-encapsulated ACA-PNIPAM-cPF127 NPs have a size range of 250-300 nm. The Zeta-Potential was measured at room temperature (~25 °C): 50 uL of the sample was diluted with 2.95 mL deionized water and then measured with Nanotrac 150 (Microtrac, Inc., Nikkiso, San Diego, CA, USA). As examples, Zeta-Potential of the ICG-encapsulated ACA- P(NIPAM-TBAm 185:15)-SDS is -16.57 ± 0.61mV, Zeta-Potential of the ICG-encapsulated ACA- P(NIPAM-TBAm 185:15)-PF98 NPs is -4.91 ± 1.53 mV, and Zeta-Potential of the ICG-encapsulated ACA-P(NIPAM-TBAm 185:15)-cPF127 NPs is -5.38 ± 0.65 mV. Both DLS and Zeta-Potential measurements confirmed the stable formation of nanoparticles.

Characterization of Carboxylized Pluronic F127

The chemical structure of synthesized carboxylized Pluronic F127 was confirmed by H^1 NMR (D_2O, 300 MHz) and ATR-FTIR.

H^1 NMR. The resonance peak at δ 2.57 ppm corresponds to the methylene protons of the succinic groups ($-CH_2-CH_2-$) added to the ends of Pluronic F127. The peaks at δ 1.04 ppm and δ 3.41–3.59 ppm were attributed to $-CH_3$ of PPO and $-CH_2CHO$ of PPO and PEO in F127, respectively.[4,5]

ATR-FTIR. The peak at 2880 cm^{-1} was attributed to the C–H stretching vibrations of PEO segments in Pluronic F127. The peak at 1080 cm^{-1} was assigned to C–O–C stretching vibration in Pluronic F127. The peak at 1740 cm^{-1} (C=O stretching) indicated that the –COOH group was successfully grafted onto the ends of Pluronic F127.[6]

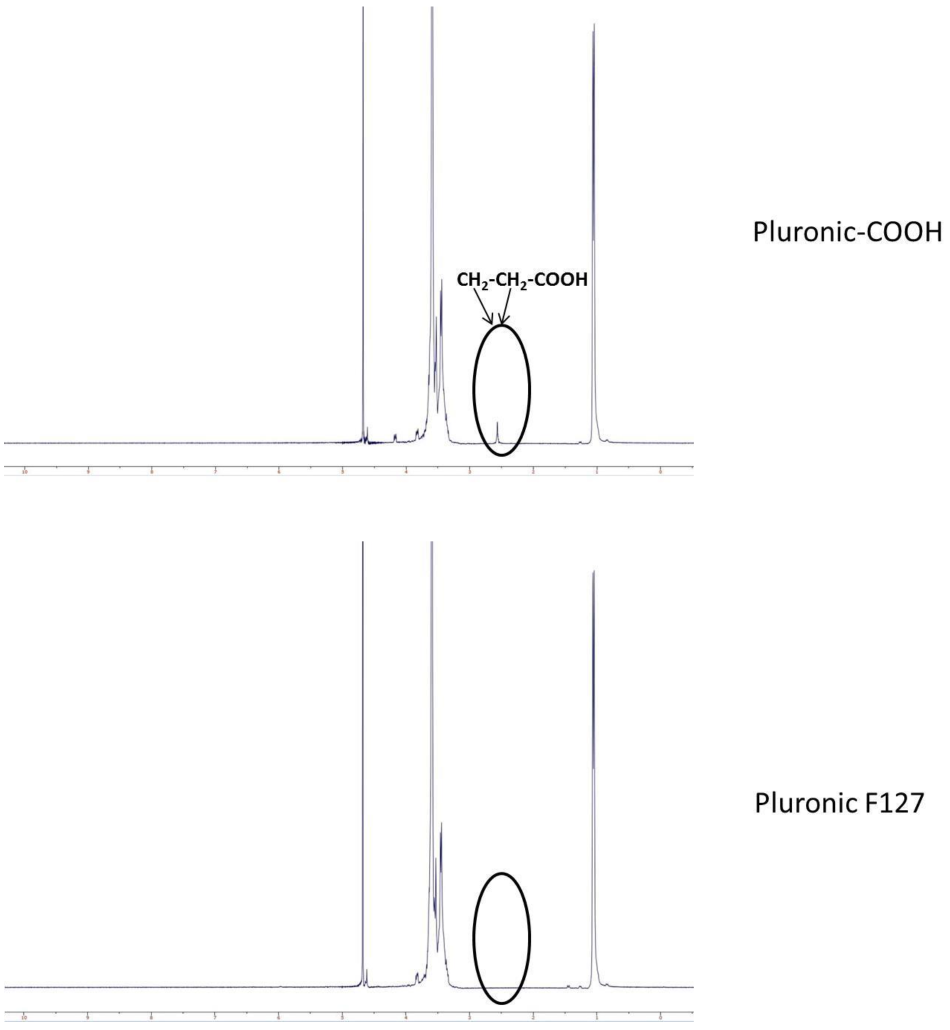

Figure S1 | 1H NMR: The newly-generated resonance peak at 2.57 ppm belongs to the hydrogens in CH2-CH2-COOH. The degree of substitution of the Pluronic-COOH is 53% (the average number of COOH per Pluronic F127).

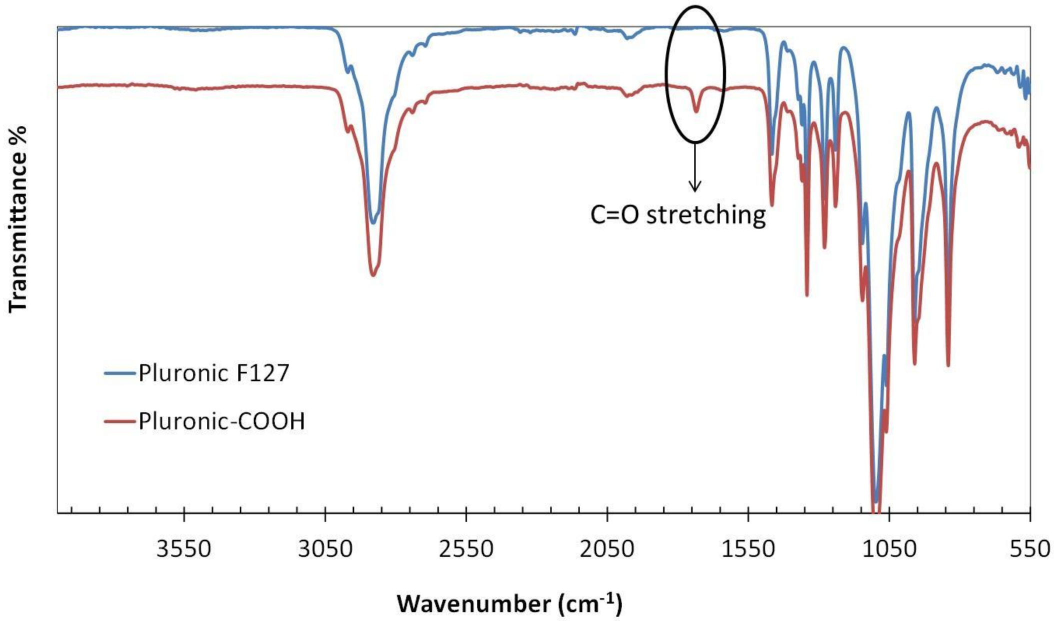

Figure S2 | FTIR: The peak at 1732 cm^{-1} corresponds to the C=O stretching in the –O–CO– and –COOH groups.

Measuring Excitation/Emission Spectrum of ICG-NPs

The excitation/emission spectrum of ICG-NPs (200 uL each sample) was measured at room temperature (~25 °C) with Infinite M200 PRO (Tecan, Inc., Morrisville, NC, USA) ICG in DI water (87.8 nmol/mL, same dye concentration as ICG-NPs) was also tested as comparison. All various ICG-NPs have similar excitation/emission spectrums. The results were shown in Figure S3 (a) and (b) correspondingly. The excitation peaks of all the ICG-NPs are located in the range of 660-720 nm, and their emission peaks are around 690 nm. In the measurement of the excitation spectra, we respectively used 830-nm and 850-nm long pass filters for the detection of the fluorescence emission of the ICG-NP samples and the ICG aqueous solution. In the measurement of the emission spectra, we respectively selected 600-nm and 675-nm excitation wavelengths for the ICG-NP samples and the ICG aqueous solution. All the data were acquired and averaged from three batch samples.

(a)

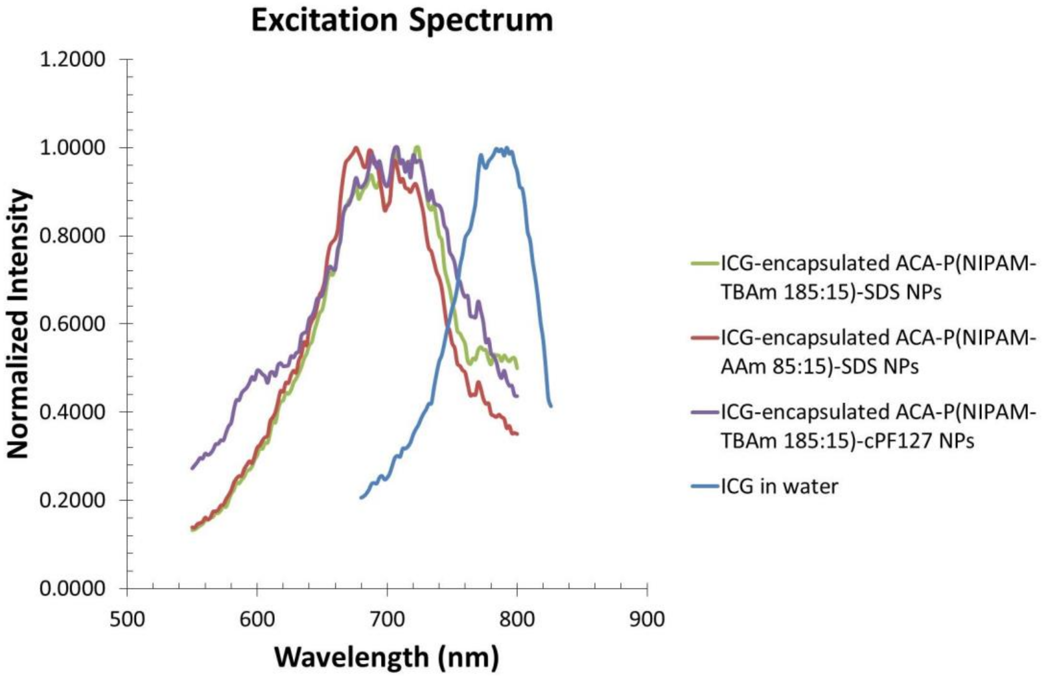

(b)

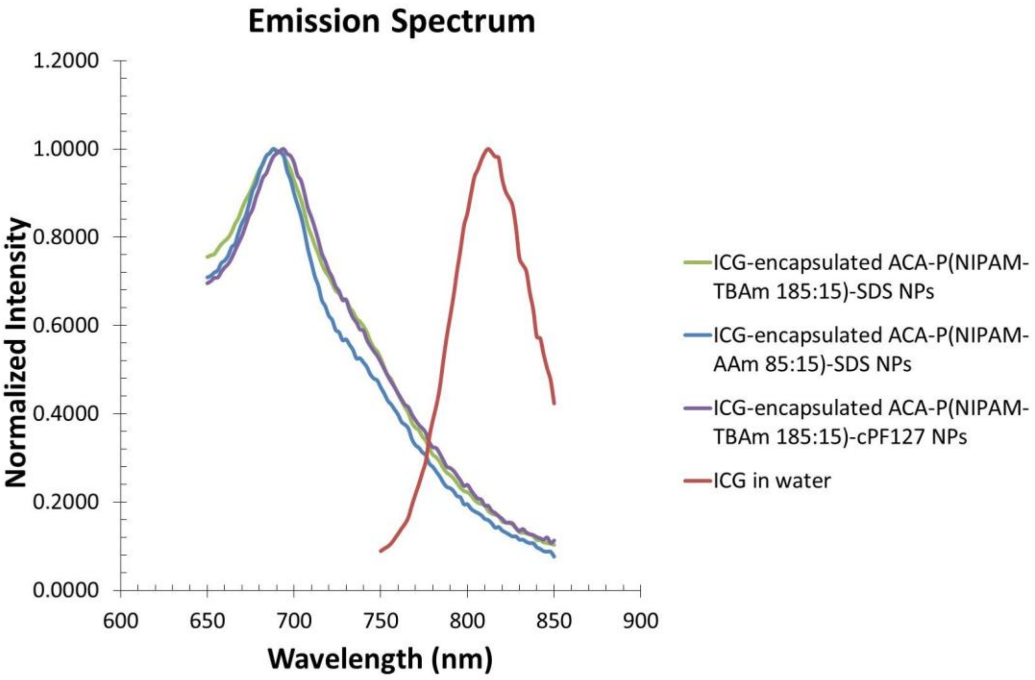

Figure S3 | Excitation and emission spectra of the ICG-NP samples and the ICG aqueous solution (87.8 nmol/mL).

References

1 Cheng, B. *et al.* Development of Ultrasound-Switchable Fluorescence Imaging Contrast Agents Based on Thermosensitive Polymers and Nanoparticles. *Selected Topics in Quantum Electronics, IEEE Journal of* **20**, 67-80 (2014).

2 Pei, Y. *et al.* High resolution imaging beyond the acoustic diffraction limit in deep tissue via ultrasound-switchable NIR fluorescence. *Sci rep* **4** (2014).

3 Cheng, B. *et al.* Centimeter-deep tissue fluorescence microscopic imaging with high signal-to-noise ratio and picomole sensitivity. *arXiv preprint arXiv:1510.02112* (2015).

4 Chen, Y., Zhang, W., Huang, Y., Gao, F. & Fang, X. Dual-functional c (RGDyK)-decorated Pluronic micelles designed for antiangiogenesis and the treatment of drug-resistant tumor. *Int J nanomed* **10**, 4863 (2015).

5 Zhang, W. *et al.* Synthesis and characterization of thermally responsive pluronic F127− chitosan nanocapsules for controlled release and intracellular delivery of small molecules. *ACS nano* **4**, 6747-6759 (2010).

6 Zhou, Q., Zhang, Z., Chen, T., Guo, X. & Zhou, S. Preparation and characterization of thermosensitive pluronic F127-b-poly (ε-caprolactone) mixed micelles. *Colloid surface B* **86**, 45-57 (2011).
